# Low-Level Laser Therapy as an Effective Intervention for Facial Pigmentation: A Case Report of Ephelides and Lentigines in Syrian Women

**DOI:** 10.1155/crdm/8197442

**Published:** 2025-08-10

**Authors:** Kawthar Shurrab, Juliana Nabil Alzghayar

**Affiliations:** Higher Institute for Laser Research and Applications, Damascus University, Damascus, Syria

**Keywords:** ephelides, freckles, lentigo, low-level laser therapy, treatment

## Abstract

Facial pigmentation disorders such as ephelides (commonly known as freckles) and solar lentigines are chronic dermatological conditions associated with sun exposure that can impact cosmetic appearance. This case report presents low-level laser therapy (LLLT) as an effective intervention for two different pigmentary disorders in Syrian women: one with ephelides and another with solar lentigines. The first case involved a 34-year-old woman with Fitzpatrick skin type II presenting with facial ephelides, while the second case featured a 49-year-old woman with Fitzpatrick skin type III diagnosed with solar lentigo. Both patients underwent 12 sessions from June 1 to August 1, 2023, using a red diode laser (660 nm) at a power density of 15.6 mW/cm^2^ and a dose of 5.6 J/cm^2^, applied for 6 min per session, with one pass per treatment area. The results demonstrated a marked reduction in pigmentation intensity and lesion size, with no reported side effects or lesion recurrence during a 1-year follow-up. These results suggest that LLLT may serve as an effective and safe therapeutic modality for selected facial pigmentary disorders.

## 1. Introduction

Low-level laser therapy (LLLT) has become increasingly relevant in dermatological aesthetics due to its capacity to influence fundamental biological responses in the skin. Through the use of specific wavelengths, particularly within the red and near-infrared spectrum, LLLT exerts noninvasive effects that support skin healing and regeneration. Conditions such as acne, inflammation, pigmentary irregularities, scars, and signs of skin aging have been shown to respond positively to this form of photobiomodulation [1].

At the cellular level, the therapy works through the absorption of light by mitochondrial photoacceptors, mainly cytochrome c oxidase, which initiates a cascade of metabolic responses. These include elevated adenosine triphosphate ATP synthesis, enhanced oxygen consumption, and the modulation of signaling pathways involved in cell repair and survival [2, 3]. Additionally, this interaction may contribute to activating endogenous stem cells, promoting natural tissue renewal [4].

Recent investigations, including works by Ngoc et al. [5] and Beigvand et al. [6], emphasize the promising role of nonablative light therapies in improving skin appearance and structure. Notably, enhancements in collagen synthesis after LLLT exposure have been documented by both Tim et al. [7] and Yang et al. [8], supporting its use in antiaging and skin revitalization. Red light in the vicinity of 633 nm and 830 nm has demonstrated effective outcomes in enhancing skin texture and promoting wound healing without reported adverse effects [9–11]. Ephelides (commonly known as freckles) and solar lentigines are distinct yet related pigmentary disorders associated with sun exposure, which have both cosmetic and psychological effects. Ephelides are flat, red or brown spots resulting from genetics and sun exposure, typically found on the face, neck, arms, and upper chest [12]. While solar lentigines are larger spots that develop in adults over 40 as a result of photodamage from prolonged sun exposure, they appear on sun-exposed areas, such as the face, forearms, and hands [13, 14]. Treating these pigmentary disorders remains clinically challenging, as conventional modalities frequently yield less than expected results and are associated with relapse or skin irritation. Recently, LLLT has gained attention for its possible role in modulating epidermal melanin activity. Studies by Lan et al. [15, 16] suggest that photobiostimulation may affect melanocyte behavior, providing a potential method for managing pigmentary conditions through cellular stimulation. Lee et al. [17] demonstrated that the melanin level decreased, and skin tone brightened after irradiation with red light during acne treatment. Galache et al. [18] reported in their review that photobiomodulation using LLLT shows promise as a treatment for melasma, but more well-designed clinical trials are needed to confirm its effectiveness. Current studies are limited and often controversial, largely due to unclear dosing, treatment sessions, and inappropriate LLLT parameters, resulting in questionable results [19, 20].

We conducted a detailed assessment of two Syrian women, ages 32 and 49, who successfully underwent LLLT sessions for facial pigmentary lesions. The treatment plan, results, and any side effects that were noticed are all covered in the report. To guarantee the best outcomes and satisfaction, we also describe the patient selection criteria. With this case report, we hope to advance clinical knowledge of LLLT as a useful, noninvasive treatment for pigmentary disorders like ephelides and solar lentigines.

## 2. Case Presentation

### 2.1. Patient History

Neither patient was on pharmacologic agents known to induce or exacerbate pigmentation, nor did they engage in traditional treatments within the past year, and there were no allergies. To avoid confounding variables, they refrained from cosmetic products throughout the laser sessions. Informed consent was obtained, and participants were counselled on strict photoprotection, including the daily use of a high-SPF broad-spectrum sunscreen. A red diode laser was applied with a wavelength of 660 nm (Konftec, Red Laser 660 nm/500 mW, Model No. emlas-650, Laser Shower), emitting a continuous wave. The power density and dose used were 15.6 mW/cm^2^ and 5.6 J/cm^2^, respectively, and the laser effective area was 32 cm^2^. The treatment began on June 1, 2023, and August 1, 2023. The protocol consisted of two sessions per week for 6 weeks. No topical anesthetics or medications were administered before, during, or after the treatment. The affected area of the face was irradiated with a laser for 6 min per session with one pass. The patient did not feel any discomfort, redness, or any side effects. Receiving treatment stopped after 12 sessions to be evaluated and follow-up was done during treatment and monthly for one year after.

The clinical features observed in both cases, including lesion distribution, morphology, patient age, and sun exposure history, were consistent with established diagnostic criteria for ephelides and solar lentigo as described in prior literature [12–14].

#### 2.1.1. Case 1

**Figure 1:**
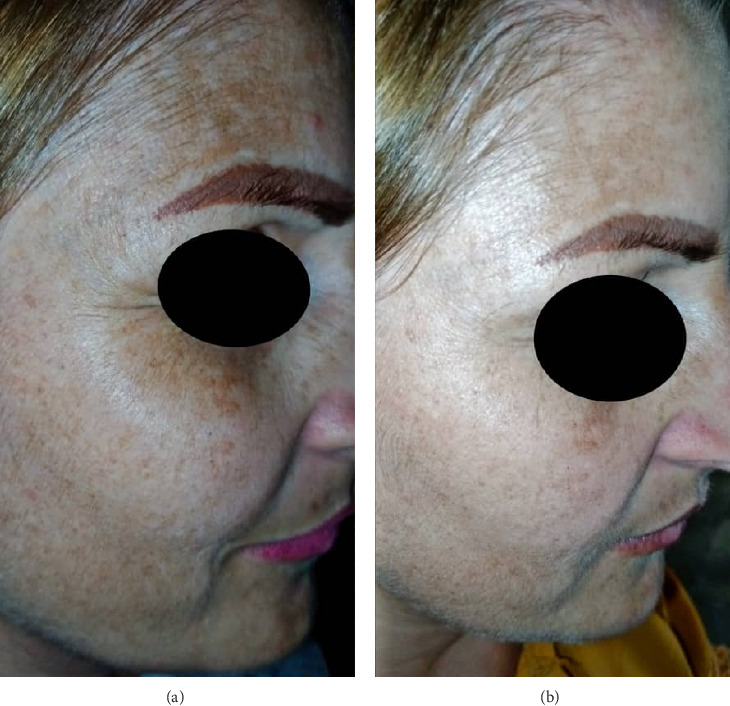
Great decrease in ephelides pigmentation was recorded at baseline and 2 months. (a) Pretreatment with LLLT. (b) 2 months after 12 sessions of treatment.

A 34-year-old Syrian woman with Fitzpatrick skin type II has multiple flat, round, and oval light-to-medium brown macules of various sizes that show no signs of inflammation. These macules are smooth, with no scaling or thickening, and tend to worsen during the summer. They are located around her eyes, on her cheeks, forehead, above her upper lip, and on her chin. She has had these ephelides on her face for the past 10 years. She did not previously receive any conventional treatment methods. The evaluation occurred two, four, and six weeks after treatment. However, assessment of the severity of ephelides by taking the photos was only available at baseline and 6 weeks post-treatment, as shown in (Figures 1(a) and 1(b)).  Figure 1(a) displays that before treatment, on the right side of the patient's face, ephelides covered all over her face.  Figure 1(b) demonstrates the treatment using LLLT after 6 weeks and 12 sessions. The patient showed a noticeable improvement in the color and lesion size of pigmentation and a more consistent tone. Additionally, there were no reports of hypopigmentation or lesion recurrence during the study.

#### 2.1.2. Case 2

**Figure 2:**
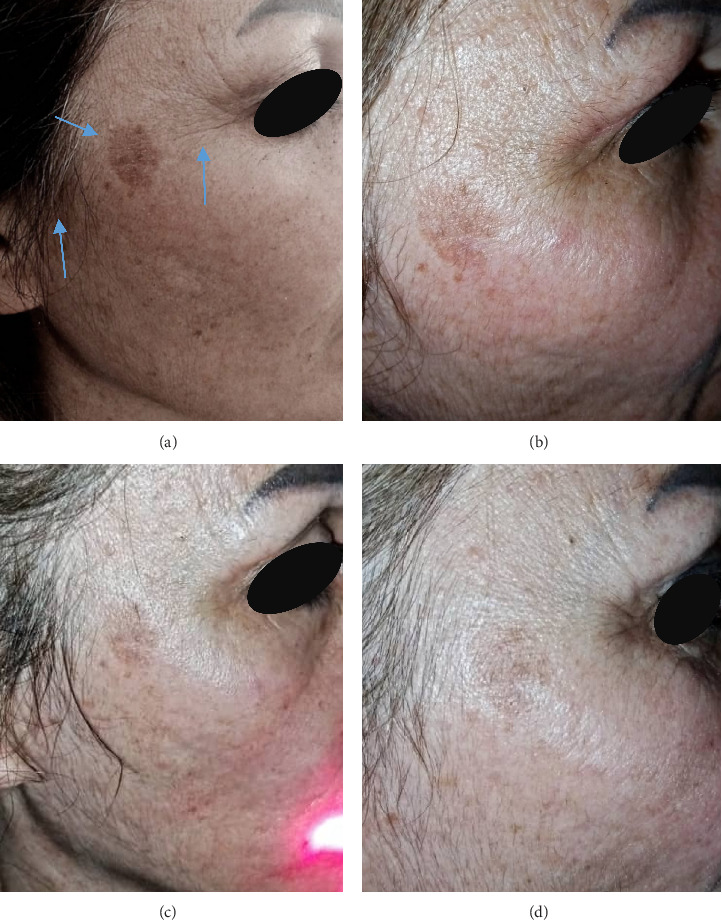
(a) Before being treated with LLLT, (b) after 2 weeks, noticeable improvement appeared, (c) after 4 weeks, and (d) after 6 weeks, a greater decrease in lentigo pigmentation, in addition to an improvement in skin overall.

A 49-year-old Syrian woman with Fitzpatrick skin type III has a hyperpigmented macule on the right malar (cheek) region. The lesion measures approximately 1.2 cm in diameter and displays a uniform light-to-medium brown pigmentation with a regular border. The surface is flat and smooth, showing no signs of scaling, ulceration, or elevation. The surrounding facial skin exhibits features of photoaging, including fine wrinkles, solar elastosis, and background mottled pigmentation, which are consistent with chronic exposure to ultraviolet (UV) radiation. These characteristics are indicative of solar lentigo. Additionally, she has had another lesion extending near the hairline bordering the right cheek for 7 years. The patient had previously received a range of conventional therapies, including topical hydroquinone, retinoids, skin-lightening creams, chemical peels, and mesotherapy. However, these interventions yielded only modest results, and the pigmentation progressively worsened over time. The severity of lentigo was assessed by taking photos before, during, and after treatment, as shown in (Figures 2(a), 2(b), 2(c), 2(d)). After treatment, the patient experienced reduced pigmentation and noted improvements in dermal texture and skin uniformity in the affected area.

Figure 2(a) displays the baseline before treatment on the right side of the patient's face. Lentigo covers a large area of her face, as there is a large patch on her cheek and next to her hairline in addition to the wrinkles around the eyes.  Figure 2(b) represents that after the treatment using LLLT for 2 weeks, the severity of lentigo decreased significantly, in addition to improving the overall skin appearance, and the wrinkles around the eyes were also remarkably reduced and became finer.  Figure 2(c) depicts the lentigo after treatment for 4 weeks, and Figure 2(d) shows that at the end of the treatment, after 6 weeks and 12 sessions, we notice a greater decrease in the size and color of pigmentation, in addition to improvement in skin tone, elasticity, and wrinkles around the eyes have become finer.

## 3. Discussion

We describe two Syrian women, aged 34 and 49, who suffered from facial pigmentation, consisting of ephelides and solar lentigines, and were successfully treated with LLLT using a continuous red diode laser with a wavelength of 660 nm. The results began to become clear compared to baseline after the fourth 2-week session, where a decrease in the intensity of pigmentation was noticed. After the eighth 4-week session, skin appearance improved, and the wrinkles became fewer and finer, with a continued improvement until the twelfth 6 week session. Follow-up was done during treatment and monthly for 1 year post-treatment. There were no negative effects, and no repigmentation was reported.

These results suggest that low-level laser exposure may affect melanin synthesis and distribution, resulting in a marked lightening of pigmented areas of the skin, which is consistent with Lan et al. [15, 16].

Both patients declared high levels of satisfaction with both the treatment process and the outcomes. They reported enhanced confidence in their appearance and expressed interest in continuing or repeating similar noninvasive treatments in the future, citing visible improvements and the absence of adverse effects.

The noticeable decrease in pigment intensity, overall enhancement in skin quality, and high patient satisfaction levels indicate that LLLT may serve as a supportive option for treating facial pigmentation concerns such as ephelides and solar lentigo.

### 3.1. Limitations

While this case report shows positive results for LLLT in improving facial skin pigmentation, there are limitations. Given that it's based on only two cases, larger statistical studies with more patients and rigorous methodologies are needed to confirm the treatment's effectiveness and compare it to other methods.

The effectiveness of the treatment was primarily evaluated through clinical observation and photographs, without the use of objective measurement tools such as the lesion size quantification or dermatologist-assessed scoring scales (e.g., MASI or mMASI for melasma), and will incorporate these in future studies. Incorporating these tools in future studies would improve the accuracy of the results and enable a quantitative assessment of any improvements.

The photographs were taken with a mobile phone camera, highlighting the need for specialized medical imaging techniques in future research.

## 4. Conclusion

This case report emphasizes the successful application of LLLT to treat ephelides and solar lentigines in two adult Syrian women with different skin tones and pigmentation types. It introduces a safe additional treatment option that could potentially benefit patients suffering from these chronic conditions, significantly enhancing their satisfaction and quality of life. Further studies comparing different wavelengths and doses, with long-term follow-up, are needed to validate these findings.

## Data Availability

Data are available from the corresponding author upon reasonable request.
